# Mapping the Risk of Fracture of the Tibia From Penetrating Fragments

**DOI:** 10.3389/fbioe.2020.544214

**Published:** 2020-09-16

**Authors:** Thuy-Tien N. Nguyen, Diagarajen Carpanen, Iain A. Rankin, Arul Ramasamy, Johno Breeze, William G. Proud, Jonathan C. Clasper, Spyros D. Masouros

**Affiliations:** ^1^Department of Bioengineering, Imperial College London, London, United Kingdom; ^2^Royal Centre for Defence Medicine, Queen Elizabeth Hospital Birmingham, Birmingham, United Kingdom; ^3^Academic Department of Trauma and Orthopaedics, Queen Elizabeth Hospital Birmingham, Birmingham, United Kingdom; ^4^Institute of Shock Physics, Department of Physics, Imperial College London, London, United Kingdom

**Keywords:** injury curve, penetrating injury, survival analysis, fracture, lower extremity, leg

## Abstract

Penetrating injuries are commonly inflicted in attacks with explosive devices. The extremities, and especially the leg, are the most commonly affected body areas, presenting high risk of infection, slow recovery, and threat of amputation. The aim of this study was to quantify the risk of fracture to the anteromedial, posterior, and lateral aspects of the tibia from a metal fragment-simulating projectile (FSP). A gas gun system and a 0.78-g cylindrical FSP were employed to perform tests on an ovine tibia model. The results from the animal study were subsequently scaled to obtain fracture-risk curves for the human tibia using the cortical thickness ratio. The thickness of the surrounding soft tissue was also taken into account when assessing fracture risk. The lateral cortex of the tibia was found to be most susceptible to fracture, whose impact velocity at 50% risk of EF1+, EF2+, EF3+, and EF4+ fracture types – according to the modified Winquist-Hansen classification – were 174, 190, 212, and 282 m/s, respectively. The findings of this study will be used to increase the fidelity of predictive models of projectile penetration.

## Introduction

Explosive devices have been the weapon of choice in recent combat and terror attacks. They were responsible for more than 60% of casualties to UK and US service personnel serving in Iraq and Afghanistan (Owens et al., 2008; Penn-Barwell et al., 2015), and were used by terrorists in civilian settings 58,095 times between 1970 and 2013, with increasing annual rate (Edwards et al., 2016). Rozenfeld et al. (2019) found that 55% of the 1,858 terrorism victims hospitalized during 1997 and 2016 were injured by explosions.

Secondary blast effects due to the penetration of energized fragments are the most common wounding mechanism in explosive events (Covey, 2002; Weil et al., 2007). Mcguire et al. (2019) studied 2,629 UK military casualties injured by explosive devices in the three recent operations (BANNER, TELIC, and HERRICK) and found that blast injury to lower extremity accounted for 35–81% of the fatal cases and 36–54% of the injured survivor. The number of injuries to the lower extremity is 20–45% in terrorist bombing attacks (Edwards et al., 2016; Rozenfeld et al., 2019). The face and lower leg are the most commonly affected body areas as observed by Breeze et al. (2015), reflecting the location of the explosive device that are commonly detonated on the ground as well as the use of personal armor covering mainly the regions of vital organs such as the thorax, abdomen, and the upper legs. Secondary blast injury by which fragments cause penetrating injury to the tibia is the most frequently observed wounding pattern in modern conflicts and associated with risk such as infection, slow recovery rate, potential amputation due to secondary complications; it can also contribute to the risk of traumatic amputation of the limbs (Khatod et al., 2003; Enninghorst et al., 2011; Davis Sears et al., 2012; Covey and Ficke, 2016; Nguyen et al., 2020). Energized fragments may be primary, such as those from the device itself, or secondary, such as debris from surrounding structures. Such projectiles are accelerated to velocities in the range of 1,000 m/s by the energy from the explosion but quickly decelerate to 600 m/s or less before penetrating the human body (Bowyer, 1996).

The majority of studies on penetrating injuries are based on case reports and field observations and are motivated by gun-shot trauma; there are limited experimental studies quantifying the risk of penetrating trauma by energized fragments, and even fewer specifically on the tibia. Hill and Watkins (2001) and Keirl et al. (2018) both used a gas gun system and animal models of red deer and pig, respectively, to impact the tibia with a small steel projectile. They, however, did not produce any injury-risk curve or suggested any scaling of the results from the animal model to the human as their aims were to verify the effect of preloading on the fracture of the tibia and the efficacy of systemic antibiotics on fracture treatment. Dougherty et al. (2011) investigated fracture to the cadaveric tibia, but with indirect impact (projectile just passing through the adjacent soft tissue) by bullets and also did not carry out a risk assessment. Experimental studies of ballistic penetrating trauma on other tissues include Kieser et al. (2014) who observed micro-fracture in deer femora by a slow velocity steel sphere using a gas gun system, Chen et al. (2016) who developed a hypervelocity (>1,000 m/s) gas-gun platform to study the local and remote effects of the spherical projectile on soft tissue and organs, and Huelke et al. (1967) and Bir et al. (2016) both of whom carried out ballistic impacts on human femora to observe the fracture patterns and to identify synthetic bone surrogates. Again, these studies did not carry out any injury-risk analysis or suggested scaling of results to the human.

Studies that quantified the injury risk to the lower leg have focused on an automotive or military vehicle injury mechanism, replicating vehicle-occupant injuries due to automotive collisions and anti-vehicle mine loads (Yoganandan et al., 1996; Mckay, 2010; Quenneville et al., 2011; Bailey et al., 2015b). The injury mechanism and pathophysiology of such loading is, however, very different to that of secondary blast injury and thus their results cannot be extrapolated to quantify the risk of secondary blast injury to the tibia. Our previous study (Nguyen et al., 2020) was the first to quantify the risk of secondary blast injury to the tibia. It proposed an experimental model of injury due to a fragment simulating projectile (FSP) using a gas-gun system and used it to generate (a) injury-risk curves of different fracture severities, and (b) a scaling method for extrapolating animal results to the human. The investigation, however, only looked at impact at the anteromedial aspect of the tibia and the scaling factor of the risk curves was based on only a limited number of samples.

In this study, we hypothesize that the severity of tibial fracture caused by a blast fragment and the injury thresholds depend on the orientation of the tibia relative to the fragment’s flight. The aim of this paper is to expand our previous work (Nguyen et al., 2020) to investigate the fracture pattern by the directional effect of FSP impact on the tibia. In addition, it generalizes the scaling approach for more representative demographics and includes the energy attenuation of the soft tissue surrounding the long bone.

## Materials and Methods

**FIGURE 1:**
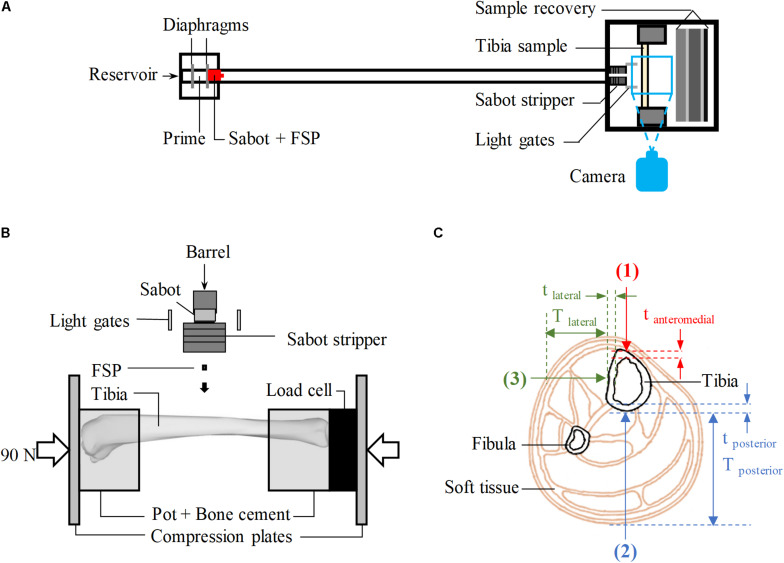
**(A)** Schematic of the gas-gun system. **(B)** Schematic of the experimental set-up inside the target chamber, viewed from above. **(C)** Cross-sectional view of the human calf showing the target locations of impact (red – anteromedial, blue – posterior, green – lateral); T_*posterior*_ and T_*lateral*_ are the thickness of soft tissue in posterior and lateral directions, respectively; t_*anteromedial*_, t_*posterior*_, and t_*lateral*_ are, respectively, the cortical of the tibia at the impacted location in anteriomedial, posterior and lateral directions.

Tests were conducted using the experimental protocol described by Nguyen et al. (2018). A schematic of the employed stainless-steel gas gun system is shown in Figure 1A where carbon-steel cylinders, 4.5 mm in diameter and 0.78 g in mass, were used as the FSP. The FSP was housed at the front of a hollow polycarbonate sabot, centred by an aluminum front plate. A 2-l reservoir charged with air or helium and a Mylar diaphragm firing mechanism were used to accelerate the sabot-FSP unit down the 3-m-long, 32-mm-bore barrel. The output velocity, between 20 and 600 m/s, could be controlled by the thickness of the diaphragm. As the sabot and FSP entered the target chamber, they were separated by the sabot stripper constructed from aluminum and polycarbonate slabs and a heavy stainless-steel block. The sabot was halted while the FSP continued to travel forward and strike the tibia sample. Layers of wood and rubber were put up at the rear of the target chamber for sample recovery. The impact speed of the FSP was measured using high-speed photography (Phantom VEO710L camera, AMETEK, United States).

Fifty-seven tibiae of skeletally mature sheep, between 36 and 80 months old, were acquired from a local abattoir and dissected out all soft tissue leaving only the periosteum intact. The tibiae were stored at −20° and used within 3 months. Mass, overall length, mid-diaphysis thickness, and mid-diaphysis cortical thickness were measured (Table 1). The ovine model was used to obtain the bulk of the results because the ovine tibia has similar geometry and material properties to the human tibia (Nguyen et al., 2020). The bones were potted in cylindrical cups with polymethylmethacrylate (PMMA) bone cement (Simplex Rapid ACR300 Autopolymerising Acrylic Resin, Swindon, United States) as shown in Figure 1B. The ovine tibia has similar size to the tibia of a 5-year-old boy (Nguyen et al., 2020), thus, axial compression of 90 ± 5 N [half the body weight of a 5-year-old boy (World Health Organisation [WHO], 2007)], measured by a 6-axis load cell (Sunrise Instruments, United States), was applied to the potted sample. This boundary condition was considered relevant, assuming that the individual would be standing at the time of injury. During the whole process, the samples were kept moist with sprayed water to prevent them from drying out.

**TABLE 1:** Average mass and dimensions of the tibiae harvested from skeletally mature sheep.

	Anteromedial^+^ (*n* = 32)	Posterior (*n* = 29)	Lateral (*n* = 28)
Mass (g)	204 ± 35	225 ± 43	185 ± 35
Maximum length (mm)	229 ± 20	243 ± 20	243 ± 12
Mid-diaphysis thickness* (mm)	15 ± 1	18 ± 2	19 ± 2
Mid-diaphysis cortical thickness** (mm)	4 ± 1	4 ± 1	4 ± 1

*Thickness of mid-diaphysis was measured as the distance between the impacted surface and the opposite surface of the tibia. **Cortical thickness was estimated from radiographs as the distance between the front and back surface of the impacted cortex. ^+^Measurements for the anteromedial cases were taken from Nguyen et al. (2020).

For all experiments, FSPs were aimed at the diaphysis which is where most below-knee traumatic amputations occur (Hull and Cooper, 1996). With the aid of a laser pointer, the samples were aligned in the target chamber to be struck by the FSP at one of the three locations on of the diaphysis shown in Figure 1C: twenty-nine tibiae were impacted at the posterior surface (direction 2), and twenty-eight tibiae were impacted at the lateral surface (direction 3). These were compared with the Results from 32 impacted tibiae on the anteromedial surface (immediately medial to the anterior border, direction 1, Figure 1C) previously reported (Nguyen et al., 2020). All experimental conditions for the impacts in directions 2 and 3 were carefully kept the same as in our previous study (Nguyen et al., 2020), including those to optimize the number of specimens used; tibiae with no fracture after an impact at a low velocity, typically below 100 m/s, were tested again at a higher velocity, typically above 200 m/s.

**FIGURE 2:**
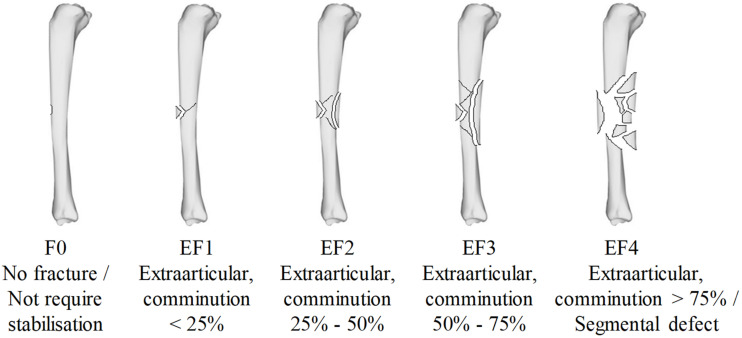
The modified Winquist-Hansen fracture classification applied to the tibia impacted by a small metallic fragment-simulating projectile; adapted from Memarzadeh et al. (2017).

Each sample underwent plain radiographic scanning using a mini C-arm (Fluoroscan InSight^TM^ FD system, United States) before testing in order to detect any existing damage and after testing in order to classify the fracture. The method was analogous to that previously described (Nguyen et al., 2020) in that two plain radiographs were taken with the impacted surface parallel and subsequently perpendicular to the imaging plane. Fracture patterns were classified by severity according to the percentage of cortical damage, using the modified Winquist-Hansen classification (Winquist and Hansen, 1980; Brito et al., 2013), independently by three trauma and orthopedic surgeons who were blinded to the impact conditions; these ranged from F0 (no fracture), EF1 (comminution < 25%), EF2 (comminution 25–50%), EF3 (comminution 50–75%), and EF4 (comminution > 75%) as shown in Figure 2.

### Statistical Analysis

Survival analysis was conducted using NCSS (v11, Utah, United States) to obtain the injury probability curves; this is an established type of analysis in injury biomechanics (Funk, 2002; McKay and Bir, 2009; Yoganandan et al., 2014, 2016a; Carpanen et al., 2019). The objective was to generate fracture-risk curves by correlating the probability of tibial fracture with FSP velocity. A likelihood-criteria best-fit analysis was used to identify the distribution that best fit the data for each fracture type at each impact location. The lognormal distribution, with the most observed highest likelihood values (Supplementary Table A), was chosen as the probability distribution most suitable for all fracture types and impact scenarios in this study.

The probability *P*(*v*) of the tibia sustaining a fracture when impacted by the FSP at velocity *v* was obtained through the lognormal regression model:

$$P\left(v\right)=\frac{1}{2}\left(1+erf\left(\frac{ln\left(v\right)-\lambda }{\kappa \sqrt{2}}\right)\right),$$

where the predictor variable is the impact velocity of the FSP, λ and κ are, respectively, the scale and shape coefficients associated with the predictor variable, and *erf* is the standard Gaussian error function. For each type of fracture produced by impacts from each direction investigated, left censoring was used if the corresponding samples sustained that fracture or a more severe one. Right censoring was used if samples sustained less severe or no fracture. Interval censoring was used for samples that underwent multiple tests.

### Scaling and Effects of Soft Tissue

Fracture-risk curves obtained from the ovine model were scaled to corresponding fracture-risk curves for the human tibia based upon recommended animal scaling parameters, where the scale is equal to the length of the parameter of the human species divided by that of the animal species used (λ_*L*_ = *L*_1_/*L*_2_) (Panzer et al., 2014). The human-ovine cortical thickness ratio was chosen as the parameter to scale the fracture-risk curves obtained; this scaling parameter has been demonstrated and validated previously (Nguyen et al., 2020).

The human fracture-risk curves were subsequently adjusted to account for the kinetic energy loss that would be expected of the FSP as it penetrates through any surrounding soft tissues, prior to impact with the bone. This adjustment was performed in accordance with conservation of energy principles. The soft tissue energy loss was calculated using previously published work of FSP impact tests into 20% by weight ballistic gelatine blocks (Nguyen et al., 2018) to determine the required energy to penetrate the block and stop at a depth of penetration equal to the expected soft-tissue thickness surrounding the injury location (anteromedial, posterior or lateral). Ballistic gelatine blocks have been used widely as a subdermal soft-tissue simulant due to their similar response to human tissue (Shepherd et al., 2009; Appleby-Thomas et al., 2011; Kieser et al., 2014; Carr et al., 2015, 2018). Furthermore, the study by Breeze et al. (2013a) comparing 20% by weight ballistic gelatine with the muscle tissue of various anatomical regions of porcine specimens has shown comparable behaviors between ballistic gelatine and the leg muscles against penetrations by cylindrical metallic FSPs.

Combining both fracture scaling and the energy loss due to penetration through soft tissue, the impact velocity of the FSP when it reaches the human leg is therefore calculated as:

$${\text{v}}_{\text{impact}}^{\left(\text{human}\right)}=\sqrt{{\left(\rho_{\text{cortical}}\times {\text{v}}_{\text{impact}}^{\left(\text{ovine}\right)}\right)}^{2}+{\text{v}}_{\text{penetration}}^{2}},$$

where ${\text{v}}_{\text{impact}}^{\left(\text{ovine}\right)}$ is the value of the predictor variable (the FSP impact velocity at the ovine tibia) for the corresponding risk of fracture obtained from the survival analysis of ovine tests, ρ_cortical_ is the human-to-ovine ratio of cortical thickness at the impacted cortex, and v_penetration_ is the impact velocity of the FSP required to penetrate into a 20% by weight ballistic gelatine block and stop at the depth of penetration equal to the soft-tissue thickness at the impact location.

Thirty-three computerised tomography (CT) scanned images (1-mm-thick 0.5 × 0.5-pixel transverse slices; Siemens Somatom Definition AS 64, Erlangen, Germany) from −20°C fresh-frozen post-mortem human subjects (PMHSs) were used to obtain the cortical thickness (t_human_) of different cortices and the corresponding soft-tissue thickness (T) (Table 2) using Mimics (v20.0.0.691, Materialise, Leuven, Belgium). For each impact direction, the thickness of the incident side at the mid-diaphysis was measured from all the scanned images to calculate the average cortical thickness of the corresponding impacted cortex. The Imperial College Tissue Bank ethics committee had granted ethical approval for use of the CT images in this study (ethical approval number: 12-WA-0196). The PMHSs used were 51 ± 6 years old, 173 ± 10 cm tall, 72 ± 15 kg, and with no known pathology affecting tissues of the limbs. Due to the horizontal positioning of each PMHS in the CT scanner, however, the posterior soft tissue was distorted rendering the corresponding thickness measured from the CT scan unreliable. Thus, the posterior soft-tissue thickness was estimated from morphological studies of the human calf and tibia (Claessens et al., 1991; Cristofolini and Viceconti, 2000; Kügler et al., 2001; Nande et al., 2008; Cuk et al., 2012).

**TABLE 2:** The average (± standard deviation) thickness of the tibial cortex and soft tissue measured from the postmortem human subjects, their corresponding cortical thickness ratio, and the required impact velocity for penetration through the soft tissue.

*n* = 33	Anteromedial	Posterior	Lateral
Tibia cortical thickness*, t_human_ (mm)	12 ± 2	6 ± 1	5 ± 1
Human-ovine cortical thickness ratio, ρ_cortical_	3	1.5	1.3
Soft-tissue thickness, T_human_ (mm)	4 ± 3	85 ± 8**	41 ± 11
v_penetration_ (m/s)	–	210	95

*Cortical thickness was estimated from CT scan as the distance between the front and back surface of the impacted cortex. **The posterior soft-tissue thickness was obtained from morphological studies (Claessens et al., 1991; Kügler et al., 2001; Nande et al., 2008; Cuk et al., 2012).

The cortical thickness ratio ρ_cortical_ = t_human_/t_ovine_ for a specific cortex was calculated using t_human_ as the relevant measured average value from the PMHSs (Table 2) and t_ovine_ as the average thickness of the ovine samples undergoing impacts on that cortex (Table 1). The required v_penetration_ corresponding to a soft-tissue thickness, T was obtained from the study by Nguyen et al. (2018) reporting the relationship between the FSP impact velocity and the depth of penetration in soft-tissue simulant.

## Results

**FIGURE 3:**
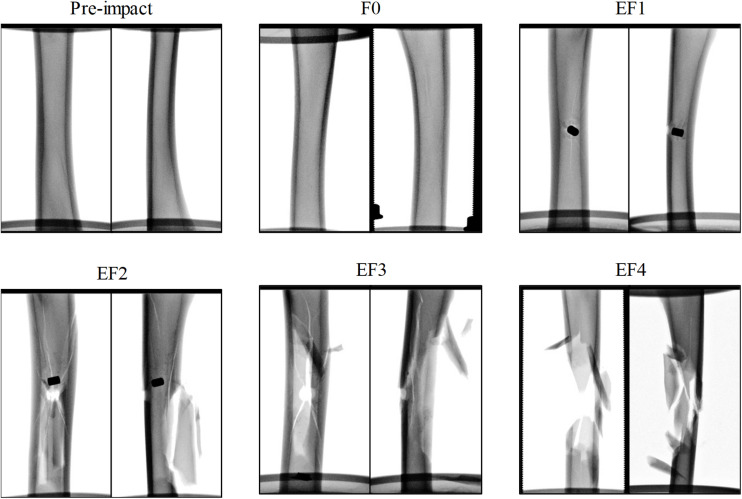
Exemplars of radiographs of the ovine tibia samples before and after impact by the projectile, classified by the mWH classification, with anterior/posterior **(left)** and lateral/medial **(right)** view.

For all impacted locations, it was observed that the fracture pattern was more severe as the FSP impact velocity increased. At velocities below 100 m/s, impacts typically did not penetrate the tibia and resulted in outer surface indentation at most; these were classified as F0. With increasing impact velocity, the fracture pattern progressed from a puncture at the impacted cortex, similar to the drill-hole fracture pattern and the FSP was strapped inside the tibia with little or no fracture presented in other cortices (classified as EF1), to a drill-hole puncture at the front and a large opening at the rear of the impact location where the FSP escaped, with butterfly pattern fractures (classified as EF2 or EF3), multiple fractures and crack lines (classified as EF3 or EF4), and comminuted fractures (classified as EF4). Examples of these fracture patterns are shown in the radiographs presented in Figure 3.

### Injury-Risk Curve for Posterior Impacts

**FIGURE 4:**
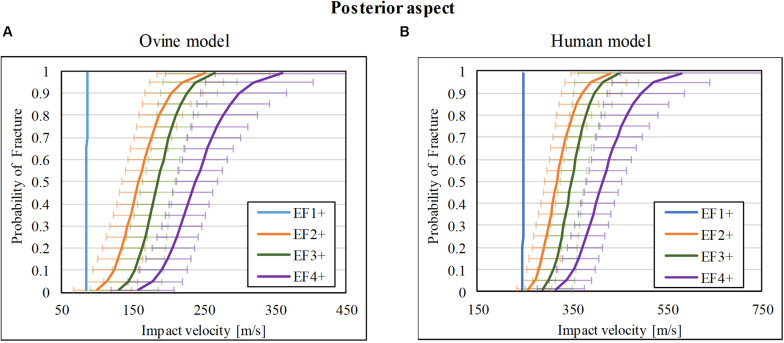
Fracture-risk curves due to projectile impact on the posterior aspect. Fracture-risk curves of EF1+ (blue), EF2+ (orange), EF3+ (green), and EF4+ (purple) fractures classified according to the mWH classification (Brito et al., 2013) for **(A)** ovine (left) and **(B)** human (right) models. The x-axis shows the predictor variable (the impact velocity of the FSP), and the y-axis shows the probability of sustaining a certain fracture. The error bars indicate 95% confidence intervals.

There was a total of 35 impact tests to the posterior cortex of the ovine tibia mid-diaphysis. 6/35 sustained no fracture (F0), 9/35 sustained EF1 fracture, 3/35 sustained EF2 fracture, 7/35 and 10/35 sustained EF3 and EF4 fractures, respectively. The risk curves for the ovine model are plotted in Figure 4A. The impact velocities of the FSP at 50% risk (V_50_) for EF1 or greater (EF1+), EF2 or greater (EF2+), EF3 or greater (EF3+), and EF4 or greater (EF4+) fractures were, respectively, 86, 159, 186, and 238 m/s.

The scaled fracture-risk curves of EF1+, EF2+, EF3+, and EF4+ fractures for the human tibia posterior cortex are shown in Figure 4B. They were obtained using $v_{impact}^{\left(human\right)}=\sqrt{{\left(1.5\times v_{impact}^{\left(ovine\right)}\right)}^{2}+210^{2}}$. The V_50_ of the projectile when it reaches the lower leg for fracture severities EF1+ to EF4+ were 246, 317, 349, and 415 m/s, respectively.

### Injury-Risk Curve for Lateral Impacts

**FIGURE 5:**
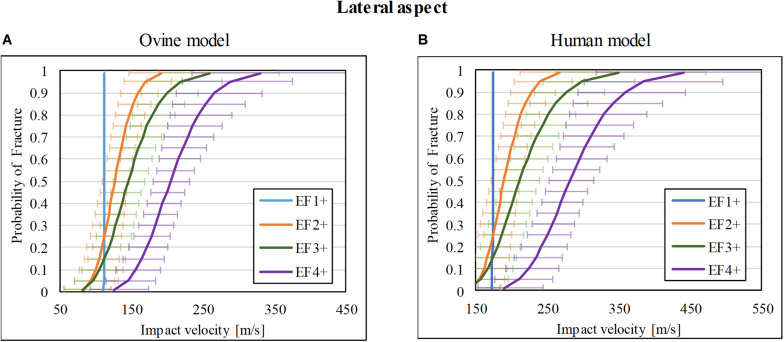
Fracture-risk curves due to projectile impacts on the lateral aspect. Fracture-risk curves of EF1+ (blue), EF2+ (orange), EF3+ (green), and EF4+ (purple) fractures classified according to the mWH classification (Brito et al., 2013) for **(A)** ovine (left) and **(B)** human (right) models. The x-axis shows the predictor variable (the impact velocity of the FSP), and the y-axis shows the probability of sustaining a certain fracture. The error bars indicate 95% confidence intervals.

Of the 37 impacts on the lateral cortex of the ovine tibia, 9/37 resulted in no fracture (F0), whereas 3/37, 4/37, 10/37, and 11/37 resulted in EF1, EF2, EF3, and EF4 fractures, respectively. The fracture-risk curves for the ovine model are shown in Figure 5A. The V_50_ of the for EF1+, EF2+, EF3+, and EF4+ fractures were 112, 127, 146, and 205 m/s, respectively.

The scaled fracture-risk curves of EF1+, EF2+, EF3+, and EF4+ fractures for the human tibia lateral cortex are shown in Figure 5B. They were obtained using ${\text{v}}_{\text{impact}}^{\left(\text{human}\right)}=\sqrt{{\left(1.3\times {\text{v}}_{\text{impact}}^{\left(\text{ovine}\right)}\right)}^{2}+95^{2}}$. The V_50_ of the projectile when it reaches the lower leg for fracture severities EF1+ to EF4+ were 174, 190, 212, and 282 m/s respectively.

### Injury-Risk Curve for Anteromedial Impacts

**FIGURE 6:**
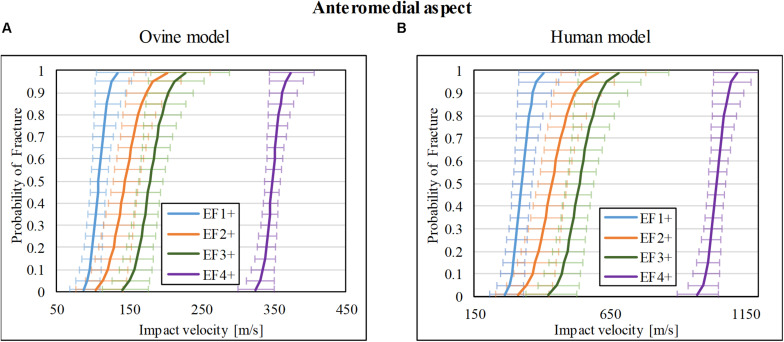
Fracture-risk curves due to projectile impacts on the anteromedial aspect. Fracture-risk curves of EF1+ (blue), EF2+ (orange), EF3+ (green), and EF4+ (purple) fractures classified according to the mWH classification (Brito et al., 2013) for **(A)** ovine (left) and **(B)** human (right) models. The x-axis shows the predictor variable (the impact velocity of the FSP), and the y-axis shows the probability of sustaining a certain fracture. The error bars indicate 95% confidence intervals.

Thirty-nine impact tests to the ovine tibia at the anteromedial mid-diaphysis were taken from our previous study (Nguyen et al., 2020), which resulted in 8/39 with no fracture (F0), 5/39 with EF1 fracture, 5/39 with EF2 fracture, 15/39 with EF3 fracture, and 6/39 with EF4 fracture. A similar number of tests with a similar range of impact velocities to those for lateral and posterior impacts were chosen from that study (Nguyen et al., 2020) for the anteromedial aspect so that the resultant fracture risk obtained here is comparable to the other two locations. The risk curves were re-analyzed and are plotted in Figure 6A for fracture types EF1+, EF2+, EF3+, and EF4+. The V_50_ for these fracture types were 108, 145, 179, and 348 m/s, respectively.

The scaled fracture-risk curves of EF1+, EF2+, EF3+, and EF4+ fractures for the human tibia anterior cortex are shown in Figure 6B. They were obtained using $v_{impact}^{\left(human\right)}=3.0\times v_{impact}^{\left(ovine\right)}$. The V_50_ of the projectile when it reaches the lower leg for fracture severities EF1+ to EF4+ were 325, 426, 457, and 1,045 m/s, respectively.

### Fracture Pattern Comparison

**FIGURE 7:**
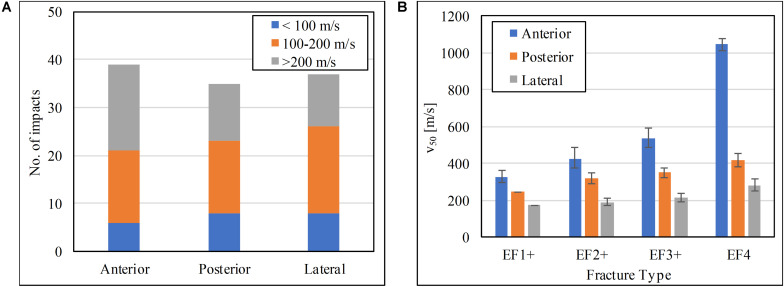
**(A)** Impact velocity distributions, and **(B)** the V_50_ impact velocity at the soft tissue for the human tibia at the three impact locations tested.

The range of impact velocity used to test in each one of the three locations on the tibia was similar (Figure 7A). The scaled V_50_ at the soft tissue surface for the human model for different fracture outcomes at the three impact locations is shown in Figure 7B. For all fracture severities, impact at the anteromedial surface of the tibia had the highest V_50_ followed by impact posteriorly, and then laterally.

## Discussion

This study is the first to report and compare the risk of fracture to the tibia when the lower leg is impacted by an FSP on its anteromedial, posterior, and lateral aspects. The FSP velocity with 50% risk (V_50_) of resulting in a specific severity of fracture (EF1+, EF2+, EF3+, or EF4) generated from impacts on these aspects were compared to each other; the lower the V_50_ value for the same fracture severity, the more vulnerable the impacted tibial aspect. Among all the impacted locations, the anteromedial surface is the least susceptible to fracture by FSP impact. This is likely because the anterior cortex is the thickest of all cortices, thus can absorb more energy from the impact; crack initiation and propagation are more suppressed compared to a thinner cortex, and so more energy is required for comminution to occur. The fracture thresholds are the lowest for the lateral surface; the lateral cortex is the thinnest and with less soft tissue compared to the posterior side; that makes this cortex the most vulnerable to fragment penetration.

These results confirm our hypothesis that the severity of tibial fracture caused by a blast fragment and the injury thresholds depend on the orientation of the tibia relative to the fragment’s flight. They are valuable for the design and assessment of personal protective equipment. The mapping of the susceptibility to injury by location can inform the requirement of protection distribution; in this case, if the same level of risk is intended, then more protection is needed on the lateral side of the lower leg, followed by the posterior and then the anterior surface. The V_50_ values of different fracture severities can inform the assessment criteria of protection for the lower leg.

The human-to-ovine cortical thickness ratio was used to scale the impact velocity from the ovine bone to the human bone and the conservation of kinetic energy was used to account for the thickness of the soft-tissue surrounding the tibia in the scaling function. The bone-to-bone scaling by the cortical thickness ratio was proposed and validated between ovine and human samples in the work by Nguyen et al. (2020). The ovine specimen was chosen as the tibia surrogate due to its availability and similarity to the human tibia. Specifically, the ovine tibia has similar material properties to that of the human tibia and its geometry is closely matching that of a 5-year-old boy (Nguyen et al., 2020). Hence, the ovine tibia can be considered representative of a 5-year-old male tibia, and a 90-N load, which is half of a 5-year-old body weight (World Health Organisation [WHO], 2007), was deemed appropriate to simulate the human standing gait. This chosen boundary condition eliminates any interspecies discrepancy in scaling due to bipedal vs. quadrupedal posture. The fracture patterns observed in ovine tibiae were also similar to those previously reported in the human tibia (Nguyen et al., 2020) as well as femur (Huelke et al., 1967; Smith and Wheatley, 1984). The cortical thicknesses obtained from the CT scans of the PMHSs (51 ± 6 years old) should be representative for young adults as bone quality has been shown to remain relatively consistent for the age group of 20–60 years (Russo et al., 2006). The cortical thicknesses of the ovine specimens were obtained from radiographs which are less accurate than CT scans. The samples were carefully orientated to minimize any spatial discrepancy, but overestimation in the ovine cortical thickness, although likely minimal, may still be present leading to an underestimation in the scaling parameter. Using the cortical thicknesses of a specific corresponding cortex for scaling, which were obtained from the tested ovine tibiae and PMHS tibiae from a representative demographic, ensured the accuracy and relevance of the scaled results; this is an important improvement from the previous study (Nguyen et al., 2020).

As previous work has shown that there are no inertial effects due to the surrounding soft tissue of the leg on the resulting severity of fracture (Nguyen et al., 2020), this study dissected out all soft tissue and tested directly on bone. The effect of the soft tissue on decelerating the projectile, however, had to be accounted for so as to adjust the fracture-risk curves to have the velocity with which the projectile reaches the as the predictor variable. This was necessary because protection is employed around the leg and therefore experiences – and so should be qualified against – the velocity with which the projectile reaches the leg. The relationship between depth of penetration in 20% ballistic gelatine and impact velocity of the FSP here has been reported previously (Nguyen et al., 2018) and was employed to make the adjustment in the fracture-risk curves. The thickness of the soft-tissue layer used for the estimation was obtained from CT scans of PMHS specimen; even though the skeletal muscle mass of the leg stays the same for adults below 60 years old (Lynch et al., 2019), there may have been volume loss due to post-mortem shrinkage of the soft tissue and thermal shrinkage during freezing and thawing. The potential underestimations in soft-tissue thickness as well as the fact that skin was treated the same as muscle tissue may have resulted in a conservative estimation of the risk of fracture.

The 0.78-g carbon steel cylindrical FSP was chosen as it is the most typical of small metallic projectiles observed clinically (Breeze et al., 2013c; Nguyen et al., 2020). The modified Winquist-Hansen classification was chosen to score the fracture outcomes as it is the most suitable classification for this type of penetrating injury to the bone (Nguyen et al., 2020). Left censoring was used in the statistical analysis for impacts that resulted in fracture as the minimum impact velocity required to generate that fracture (i.e., time to failure) could not be measured; this is known to be a conservative statistical approach (Breeze et al., 2013b; Yoganandan et al., 2016a). Interval censoring was used to account for the reuse of uninjured samples after one low velocity impact; reusing samples helps optimizing the number of samples required, accounting for 20% of all tests in this study. The verification of no fracture prior to reuse of a sample was carried out using plain radiographs; thus, any microfracture, if present, would not have been detected. The presence of microfractures, however, was not expected to have a big influence on the overall resultant fracture risk as shown in studies on dynamic loading of the tibia (Yoganandan et al., 1996, 2016b; Bailey et al., 2015a).

Using a similar number of impacts across the range of velocities at each impact location ensured a fair comparison of fracture thresholds between impacts at the three locations of the tibial diaphysis. The ovine risk curves generated from anteromedial impacts obtained in Nguyen et al. (2020) were very similar to those reported in the original study even though only tests with impact velocities within the range examined here were used. This suggests that the excluded data did not affect the survivability analysis.

The obtained risk curves are generally good fits to the experimental data as shown by the calculated normalized confidence interval size (NCIS) in Supplementary Tables B–D. They are also similar between different impact locations: EF1+ curves either fitted by a step function due to a diverged fitting solution or with very narrow confidence intervals, EF2+ and EF3+ curves are very close to each other with overlapping confidence intervals, and EF4+ curves have a clear distinction with the lower fracture-severity risk curves. This, together with the similarity in the observed fracture patterns, suggests that the fracture mechanism – as discussed by Nguyen et al. (2020) – is independent to the impacted direction. When the tibia is penetrated by a low velocity FSP, the drill-hole fracture is formed due to shear stress and adiabatic shear bands. As the impact velocity increases, the increased pre-impact energy produces additional cracks and fragmentations. Cavitation effects develop in the bone marrow, resulting in a large exit hole at the cortex opposite the impacted aspect and a butterfly fracture and comminution. Due to the complexity of the fracture mechanism and crack propagation as well as the micro-structural variation of each specimen, the response of the bone to the FSP varies more greatly at even higher impact velocities, leading to larger confidence intervals.

The soft tissue was considered insignificant for impacts at the anteromedial side, due to its small thickness, and was not accounted for in the scaling of the corresponding results. In the other two locations examined, however, calculations based on responses of ballistic gelatine were carried out, which resulted in an average velocity reduction of 14% in the lateral side and 43% in the posterior side; this is equivalent to an average kinetic energy attenuation (or absorbed) by the soft tissue of 26 and 68%, respectively. This suggests that when considering the risk of fracture to the bone tissue, the cortical thickness is the most important factor. To estimate the overall injury to the lower leg, however, injury to the soft tissue such as the wound depth and amount of non-recoverable soft tissue need to be taken into account as they can lead to risks such as infection, compartment syndrome, nerve damage, and rhabdomyolysis, to name but a few.

The authors recognize that these results are relevant for the single impact from the chosen FSP. Impact by other shape and size fragments, as well as impact by multiple fragments are likely in a real scenario and so should also be investigated. In addition, the effect of the subdermal soft tissue was considered only in terms of energy attenuation. The physical presence of the soft tissue may help in recovering some bone fragments during surgery, resulting in less severe fracture outcome. Furthermore, skin tissue was represented by ballistic gelatine in the energy attenuating calculation. Whilst skin has different material properties to muscle and its contribution to energy attenuation slightly underestimated, the effect of this simplification in the injury model is likely to be minimal and is outweighed by the benefits it brought to the efficiency of the experimental study. Finally, the predictor variable for the survival analysis was set as the impact velocity of the FSP because this parameter is the metric of assessment for the protective performance of body armors. For future studies where a variety of FSPs are employed, other variables such as energy, momentum, and cross-sectional area, to name but a few, can also be considered as the predictor variables, or as co-variates in the calculation of fracture-risk curves.

## Conclusion

This study identified and compared the risk of different fracture severities to the tibia when impacted by a 0.78 g cylindrical steel FSP from anteromedial, lateral, and posterior directions. The ovine model was used to obtain the risk curves, which were then scaled to the human tibia. The human-to-ovine cortical thickness ratio and conservation of energy were employed in the scaling process. The lateral aspect of the tibia was the most vulnerable to fragment penetration, followed by the posterior cortex, and the anteromedial aspect. These results can be used to predict the risk of fracture from a blast fragment, and to assist the designing process of personal protective equipment.

## Data Availability Statement

All datasets generated for this study are included in the article/Supplementary Material.

## Author Contributions

SM, JC, and WP conceived the project idea which was developed into an experimental model by T-TN. T-TN and DC carried out all the tests, including preparation of samples, and data acquisition. T-TN, IR, JC, AR, JB, and SM conducted the data analysis. T-TN drafted the manuscript. All authors contributed to the article and approved the submitted version.

## Conflict of Interest

The authors declare that the research was conducted in the absence of any commercial or financial relationships that could be construed as a potential conflict of interest.
