# Supplementary material for: Mapping the Risk of Fracture of the Tibia From Penetrating Fragments
Source: Front Bioeng Biotechnol. 2020 Sep 16;8:544214. doi: 10.3389/fbioe.2020.544214 (PMC7525181; doi:10.3389/fbioe.2020.544214)
Supplement: Supplementary file 1 [file Table_1.DOCX]

Supplementary Material

| Table 3 – Summary of survival analysis output for impacts on anteromedial surface of ovine tibiae including the normalised confidence interval size (NCIS) and quality index | | | | | | |
| --- | --- | --- | --- | --- | --- | --- |
| Fracture | Risk level | Impact velocity (m/s) | | | NCIS | Quality Index |
|  |  | Lower bound | Mean | Upper bound |  |  |
| EF1+ | 0.05 | 77.5 | 92.7 | 110.9 | 0.36 | Good |
|  | 0.1 | 82.3 | 95.9 | 111.8 | 0.31 | Good |
|  | 0.25 | 90.3 | 101.6 | 114.3 | 0.24 | Good |
|  | 0.5 | 97.5 | 108.3 | 120.4 | 0.21 | Good |
|  | 0.75 | 101.6 | 115.5 | 131.3 | 0.26 | Good |
|  | 0.9 | 103.4 | 122.3 | 144.8 | 0.34 | Good |
|  | 0.95 | 104 | 126.6 | 154.1 | 0.40 | Good |
| EF2+ | 0.05 | 90 | 114.9 | 146.5 | 0.49 | Good |
|  | 0.1 | 98.1 | 121 | 149.2 | 0.42 | Good |
|  | 0.25 | 112.3 | 131.9 | 154.8 | 0.32 | Good |
|  | 0.5 | 128 | 145.2 | 164.6 | 0.25 | Good |
|  | 0.75 | 140.3 | 159.8 | 182 | 0.26 | Good |
|  | 0.9 | 147.7 | 174.2 | 205.5 | 0.33 | Good |
|  | 0.95 | 151.1 | 183.5 | 222.9 | 0.39 | Good |
| EF3+ | 0.05 | 127.7 | 151.3 | 179.2 | 0.34 | Good |
|  | 0.1 | 136.1 | 157 | 181.2 | 0.29 | Good |
|  | 0.25 | 150 | 167.1 | 186.2 | 0.22 | Good |
|  | 0.5 | 163.1 | 179.1 | 196.8 | 0.19 | Good |
|  | 0.75 | 171 | 192 | 215.7 | 0.23 | Good |
|  | 0.9 | 175.1 | 204.4 | 238.6 | 0.31 | Good |
|  | 0.95 | 176.9 | 212.2 | 254.6 | 0.37 | Good |
| EF4+ | 0.05 | 312.6 | 331.2 | 350.9 | 0.12 | Good |
|  | 0.1 | 319.1 | 334.8 | 351.4 | 0.10 | Good |
|  | 0.25 | 329.3 | 341.1 | 353.3 | 0.07 | Good |
|  | 0.5 | 337.9 | 348.2 | 358.8 | 0.06 | Good |
|  | 0.75 | 341.9 | 355.4 | 369.5 | 0.08 | Good |
|  | 0.9 | 343.3 | 362.1 | 381.9 | 0.11 | Good |
|  | 0.95 | 343.7 | 366.1 | 390 | 0.13 | Good |

| Table 4 – Summary of survival analysis output for impacts on posterior surface of ovine tibiae including the normalised confidence interval size (NCIS) and quality index | | | | | | |
| --- | --- | --- | --- | --- | --- | --- |
| Fracture | Risk level | Impact velocity (m/s) | | | NCIS | Quality Index |
|  |  | Lower bound | Mean | Upper bound |  |  |
| EF2+ | 0.05 | 84 | 114.6 | 156.4 | 0.63 | Fair |
|  | 0.1 | 94.2 | 123.2 | 161 | 0.54 | Fair |
|  | 0.25 | 113.2 | 138.9 | 170.3 | 0.41 | Good |
|  | 0.5 | 135.8 | 158.7 | 185.4 | 0.31 | Good |
|  | 0.75 | 155.2 | 181.3 | 211.8 | 0.31 | Good |
|  | 0.9 | 167.9 | 204.4 | 248.9 | 0.40 | Good |
|  | 0.95 | 174 | 219.6 | 277.1 | 0.47 | Good |
| EF3+ | 0.05 | 109.8 | 144.6 | 190.3 | 0.56 | Fair |
|  | 0.1 | 121.1 | 152.8 | 192.7 | 0.47 | Good |
|  | 0.25 | 141.7 | 167.5 | 198.1 | 0.34 | Good |
|  | 0.5 | 164.7 | 185.6 | 209.1 | 0.24 | Good |
|  | 0.75 | 181.1 | 205.6 | 233.4 | 0.25 | Good |
|  | 0.9 | 188.9 | 225.4 | 269.1 | 0.36 | Good |
|  | 0.95 | 192 | 238.2 | 295.6 | 0.43 | Good |
| EF4+ | 0.05 | 144.3 | 178 | 219.5 | 0.42 | Good |
|  | 0.1 | 159 | 189.8 | 226.7 | 0.36 | Good |
|  | 0.25 | 184.8 | 211.5 | 242 | 0.27 | Good |
|  | 0.5 | 211.3 | 238.4 | 269 | 0.24 | Good |
|  | 0.75 | 231.2 | 268.7 | 312.4 | 0.30 | Good |
|  | 0.9 | 245.1 | 299.3 | 365.6 | 0.40 | Good |
|  | 0.95 | 252.6 | 319.3 | 403.6 | 0.47 | Good |

| Table 5 – Summary of survival analysis output for impacts on lateral surface of ovine tibiae including the normalised confidence interval size (NCIS) and quality index | | | | | | |
| --- | --- | --- | --- | --- | --- | --- |
| Fracture | Risk level | Impact velocity (m/s) | | | NCIS | Quality Index |
|  |  | Lower bound | Mean | Upper bound |  |  |
| EF2+ | 0.05 | 70.2 | 94.5 | 127.2 | 0.60 | Fair |
|  | 0.1 | 78 | 100.8 | 130.3 | 0.52 | Fair |
|  | 0.25 | 92.5 | 112.3 | 136.5 | 0.39 | Good |
|  | 0.5 | 109.8 | 126.7 | 146.2 | 0.29 | Good |
|  | 0.75 | 125.2 | 142.9 | 163.1 | 0.27 | Good |
|  | 0.9 | 135 | 159.2 | 187.8 | 0.33 | Good |
|  | 0.95 | 139.5 | 169.9 | 206.8 | 0.40 | Good |
| EF3+ | 0.05 | 71.3 | 96.9 | 131.7 | 0.62 | Fair |
|  | 0.1 | 81.6 | 106.1 | 137.9 | 0.53 | Fair |
|  | 0.25 | 101.3 | 123.3 | 150 | 0.39 | Good |
|  | 0.5 | 125.7 | 145.7 | 168.8 | 0.30 | Good |
|  | 0.75 | 148 | 172.1 | 200.3 | 0.30 | Good |
|  | 0.9 | 164.1 | 200.1 | 243.9 | 0.40 | Good |
|  | 0.95 | 172.7 | 218.9 | 277.4 | 0.48 | Good |
| EF4+ | 0.05 | 115.1 | 145.6 | 184.2 | 0.47 | Good |
|  | 0.1 | 129.1 | 157 | 190.9 | 0.39 | Good |
|  | 0.25 | 154.4 | 178 | 205.1 | 0.28 | Good |
|  | 0.5 | 181 | 204.6 | 231.3 | 0.25 | Good |
|  | 0.75 | 200.3 | 235.2 | 276.3 | 0.32 | Good |
|  | 0.9 | 213.6 | 266.7 | 333 | 0.45 | Good |
|  | 0.95 | 220.9 | 287.5 | 374.1 | 0.53 | Fair |
